# Efficacy of Kuntai capsules with low-dose Femoston for perimenopausal syndrome: a meta-analysis

**DOI:** 10.3389/fmed.2026.1768247

**Published:** 2026-06-24

**Authors:** Xiangnan Chen, Yu Wang, Baichao Shi, Jiumei Xie, Xiaoke Wu

**Affiliations:** 1 Graduate School, Heilongjiang University of Chinese Medicine, Harbin, Heilongjiang, China; 2 The First Affiliated Hospital, Heilongjiang University of Chinese Medicine, Harbin, Heilongjiang, China

**Keywords:** clinical efficacy, Kuntai capsule, Low-dose Femoston, meta-analysis, perimenopausal syndrome

## Abstract

**Background:**

Kuntai capsule (KTC) has been widely used to treat perimenopausal syndrome (PMS). However, evidence on its combination with low-dose Femoston (FMT) remains insufficient. This meta-analysis aimed to compare the clinical efficacy and safety of KTC plus low-dose FMT versus low-dose FMT alone in the treatment of PMS.

**Methods:**

Eight databases were searched from inception to October 30, 2025, for randomized controlled trials (RCTs) comparing KTC combined with low-dose FMT versus low-dose FMT alone in the treatment of PMS. Two reviewers independently screened studies, extracted data, and assessed bias risk with the RoB 1.0 tool. Meta-analyses were performed using RevMan 5.4 and Stata 15.0. The robustness of the results was evaluated by trial sequential analysis (TSA), and the certainty of the evidence was assessed using the GRADE approach.

**Results:**

Ten RCTs comprising 1,057 participants were included. Compared with low-dose FMT alone, adjunctive KTC significantly improved the clinical response rate [*RR* = 1.17, 95% CI (1.10, 1.23)] and reduced Kupperman scores [*MD* = −4.36, 95% CI (−4.93, −3.79)]. For hormonal outcomes, the combination therapy increased E_2_ levels [*SMD* = 1.06, 95% CI (0.59, 1.54)] and decreased FSH [*SMD* = −0.95, 95% CI (−1.48, −0.43)] and LH [*SMD* = −1.40, 95% CI (−2.19, −0.60)]. Regarding lipid profiles, TC, TG, LDL-C, and HDL-C were all improved in the combination group (all *P* < 0.01). The incidence of adverse events was lower in the combination group [*RR* = 0.52, 95% CI (0.33, 0.81)], and no significant increase in endometrial thickness was observed [*MD* = 0.09, 95% CI (−0.85, 1.03)]. TSA confirmed the robustness of the clinical response rate and safety findings. The certainty of evidence assessed by GRADE was moderate for the clinical response rate, Kupperman score, and HDL-C, but low or very low for the remaining outcomes.

**Conclusion:**

Based on current evidence, KTC combined with low-dose FMT may have potential advantages in improving PMS-related symptoms, sex hormone levels, and lipid profiles, with an acceptable short-term safety profile in patients. However, the certainty of evidence across the outcome measures, as assessed by GRADE, was not high. Therefore, these findings should be interpreted with caution, and further verification through high-quality, large-sample randomized controlled trials is warranted.

**Systematic review registration:**

https://inplasy.com/inplasy-2025-11-0002/, identifier INPLASY2025110002.

## 1 Introduction

Perimenopause is a natural transitional stage for women aged 45∼55 years, marking the shift from the reproductive period to old age. The World Health Organization (WHO) defines the menopausal transition as the period that begins with the onset of menopause-related symptoms and ends 1 year after the final menstrual period (1).

During this transitional phase, the gradual decline of ovarian function and the subsequent fluctuations or decreases in sex hormone levels lead to a variety of physical and psychological symptoms, which are collectively referred to as perimenopausal syndrome (PMS) (2). The clinical presentation of PMS encompasses a range of symptoms—menstrual irregularities, vasomotor symptoms, sleep disturbances, vaginal dryness, and dyspareunia—which not only reduce women’s quality of life but may also elevate the long-term risks of osteoporosis and cardiovascular disease (3). Studies show that approximately 33.9% of perimenopausal women experience depression (4), and the risks of cardiovascular disease and bone disorders are 8.88 and 24.3%, respectively (5). Given the accelerating global population aging, it is estimated that over 120 million women will be in the perimenopausal or postmenopausal stage by 2030 (6). This makes perimenopause a significant public health concern that deserves increased attention.

Hormone replacement therapy (HRT) is an effective treatment for PMS in modern medicine. Femoston (FMT), a fixed-dose combination of estradiol and dydrogesterone, is a commonly used regimen with proven efficacy in alleviating perimenopausal symptoms (7). A meta-analysis has shown that FMT is superior to traditional hormone replacement therapy in terms of certain adverse reactions. However, the potential long-term risks of HRT (e.g., venous thromboembolism, stroke, and breast cancer) remain a major concern for patients (8) and contribute to poor treatment adherence and low healthcare-seeking rates (7). Hence, exploring safe and effective alternative or combination strategies is of significant clinical importance.

Kuntai capsule (KTC) is derived from the classic Chinese formula Huanglian Ejiao Decoction. Modern research indicates that KTC can alleviate PMS-related symptoms through multiple mechanisms, including regulating ovarian function, improving vasomotor function, modulating lipid metabolism, and exerting anti-inflammatory effects, among others (9). In recent years, the combination of KTC and HRT has been applied in conditions associated with ovarian function decline, including premature ovarian insufficiency and diminished ovarian reserve (10, 11). However, no systematic review has specifically evaluated the efficacy and safety of KTC combined with low-dose FMT (estradiol/dydrogesterone) for the treatment of PMS. Therefore, this study conducted a systematic review and meta-analysis of published randomized controlled trials to assess the clinical value of KTC combined with low-dose FMT in the management of PMS, aiming to provide evidence-based support for integrative traditional Chinese and Western medicine treatment.

## 2 Materials and methods

The methodological procedures of this meta-analysis followed the Preferred Reporting Items for Systematic Reviews and Meta-Analyses (PRISMA) guidelines (12). The protocol was registered with INPLASY (INPLASY2025110002).

### 2.1 Search strategy

We conducted a systematic search of the following databases for randomized controlled trials investigating the combination of Kuntai capsule and low-dose Femoston for the treatment of perimenopausal syndrome: PubMed, Cochrane Library, Embase, Web of Science, China National Knowledge Infrastructure (CNKI), Chinese Biomedical Literature Database (CBM), China Wanfang Data, and VIP Journal Database (VIP). The search covered the period from the inception of each database to October 30, 2025. The Chinese search terms included “perimenopausal syndrome,” “perimenopause,” “menopause,” “climacteric,” “Kuntai capsule,” “Femoston,” and “randomized.” The procedure used to retrieve data from English databases is exemplified by the PubMed search strategy: (((“Perimenopause” [Mesh]) OR (((Perimenopause[Title/Abstract]) OR (Climacteric[Title/Abstract])) OR (Menopause[Title/Abstract]))) AND (Kuntai capsule[Title/Abstract])) AND ((Femoston[Title/Abstract]) OR (Estradiol[Title/Abstract] AND Dydrogesterone Tablets[Title/Abstract]))) AND ((“Randomized Controlled Trial” [Publication Type]) OR (((random[Title/Abstract]) OR (blind[Title/Abstract])) OR (random*[Title/Abstract]))). A combination of subject and free-text terms was used to search all databases. The detailed search strategies for each database are presented in Supplementary material (Search strategies).

### 2.2 Inclusion criteria

1. Participants: Participants were women with a confirmed clinical diagnosis of PMS. Western medicine diagnostic criteria were based on the ninth edition of *Obstetrics and Gynecology* (13), the 2011 International Menopause Society guidelines (14), or other authoritative diagnostic references. Traditional Chinese medicine (TCM) diagnostic criteria followed *TCM Gynecology* (15). No restrictions were imposed on age, disease duration, or symptom severity.

2. Intervention and control:

(1) The experimental group received KTC combined with low-dose FMT as the primary intervention. ① KTC (0.5 g/capsule; Guiyang Xintian Pharmaceutical Co., Ltd., China; approval no. Z20000083) was administered orally at four capsules three times daily (6 g/day). ② FMT (estradiol/dydrogesterone sequential pack, 1 mg: 10 mg; Abbott Biologicals B.V.) was given as: one white tablet (estradiol 1 mg) daily for 14 days, then one gray tablet (estradiol 1 mg + dydrogesterone 10 mg) daily for the next 14 days.

(2) The control group received the same FMT regimen without KTC.

(3) Minimum treatment duration: 4 weeks.

3. Outcomes: Eligible studies were required to include at least one of the following outcome measures.

(1) Primary outcome measure: Kupperman score; (2) Secondary outcome measures: ① Sex hormone levels: estradiol (E_2_), follicle-stimulating hormone (FSH), luteinizing hormone (LH); ② Lipid profile: total cholesterol (TC), triglycerides (TG), low-density lipoprotein cholesterol (LDL-C), high-density lipoprotein cholesterol (HDL-C); ③ Endometrial thickness; ④ Clinical response rate (treatment efficacy evaluated based on the reduction in Kupperman score); ⑤ Adverse events (e.g., breast tenderness, gastrointestinal reactions, neurological or reproductive system symptoms).

4. Study design: Randomized controlled trials (RCTs) investigating KTC combined with low-dose FMT for the treatment of PMS, regardless of the publication language.

### 2.3 Exclusion criteria

Studies meeting any of the following criteria were excluded.

(1) Non-randomized controlled trials, including those without a parallel control group or those using only self-controlled or historical controlled designs.

(2) Non-clinical studies, such as animal studies or *in vitro* studies.

(3) Unclear interventions in either the treatment or control group, e.g., failure to explicitly report the dosage of KTC or FMT; use of the conventional-dose FMT (2 mg estradiol/10 mg dydrogesterone); or the addition of other pharmacological or non-pharmacological therapies (e.g., placebo, dydrogesterone, estradiol valerate, progynova, acupuncture, etc.) in either group.

(4) Data were incomplete, or there were serious errors in the data or measurement units, or obvious logical contradictions between the reported outcome data and the final conclusions, without access to the raw data from the corresponding author.

(5) Duplicate publications.

(6) Dissertations, reviews, conference abstracts, systematic reviews, as well as study for which the full text was unavailable.

### 2.4 Literature screening and data extraction

Two reviewers conducted the literature screening and data extraction independently. First, we used Note Express to remove duplicate entries, then evaluated the selected studies based on the inclusion and exclusion criteria. In the second phase, the reviewers used a pre-structured Excel spreadsheet to extract data, including details such as the first author, publication year, patient age, disease duration, treatment duration, interventions, and relevant outcome measures. Any disagreements that arose during the process were resolved through discussion between the reviewers. When consensus could not be reached, a third reviewer made the final decision.

### 2.5 Assessment of literature quality

The Cochrane Risk-of-Bias Tool (RoB 1.0) was used to evaluate the methodological quality of the included studies (16). The evaluation focused on six elements: random sequence generation, allocation concealment, performance bias (blinding of participants and personnel), detection bias (blinding of outcome assessment), completeness of outcome data, selective reporting bias, and other potential sources of bias. The risk classification for each outcome was rated as low risk, high risk, or unclear risk.

### 2.6 Statistical analysis

Statistical analyses were performed using Review Manager Version 5.4.1 and Stata 15.0. For dichotomous variables, the results are presented as the risk ratio (*RR*) and its 95% confidence interval (*CI*). For continuous variables, when the measurement methods or units were consistent, the mean difference (*MD*) and its 95% CI were reported; when the measurement methods or units differed, the standardized mean difference (*SMD*) was used as the effect size and reported with its 95% *CI*. Although the units for LH and FSH are metrically equivalent (1 IU/L = 1 U/L = 1 mIU/mL), the included studies utilized heterogeneous assay methodologies—namely, chemiluminescence immunoassay, enzyme-linked immunosorbent assay, and radioimmunoassay—which may introduce systematic measurement bias. Therefore, *SMD* were selected as the primary effect size for both FSH and LH. Heterogeneity across studies was assessed using the I^2^ statistic. When *P* ≥ 0.10 and *I*^2^ < 50%, indicating good homogeneity, a fixed-effects model was applied. When *P* < 0.10 or *I*^2^ ≥ 50%, indicating substantial heterogeneity, a random-effects model was applied. *P* < 0.05 was considered statistically significant.

(1) Subgroup analysis. Prespecified subgroup analyses were performed for outcomes with substantial heterogeneity (*I*^2^ ≥ 50%) to explore potential sources of heterogeneity. Subgroup analyses were stratified according to mean age (≥ 50 years vs. < 50 years) and treatment duration (≥ 3 months vs. < 3 months). Because individual patient data were not available, the mean age at baseline of the included studies was used as the basis for age stratification. Between-subgroup differences were formally tested using the χ^2^ test for interaction.

(2) Sensitivity analyses: A leave-one-out sensitivity analysis was performed to assess the robustness of the pooled estimates. Specifically, each study was sequentially excluded, and the meta-analysis was repeated to evaluate whether any single study had an influence on the overall results.

(3) Publication bias. Publication bias was assessed using funnel plots and Egger’s test for outcomes with at least 10 included studies. Egger’s test with *P* > 0.05 indicated no significant publication bias. These analyses were performed in Stata 15.0.

(4) Trial sequential analysis (TSA). Trial sequential analysis was performed for dichotomous outcomes using TSA software (version 0.9.5.10 beta; available at https://ctu.dk/tools). The relative risk reduction (RRR) and the event rate in the control group were used as effect measures. The control group event rate was calculated as the number of events in the control group divided by the total number of subjects in the control group. The experimental group event rate was defined as the number of events in the treatment group divided by the total number of subjects in the treatment group. RRR was defined as (control group event rate—experimental group event rate)/control group event rate. Parameters were configured according to the methodology described by Wetterslev et al. (17): the required information size (RIS) was set as the accrued sample size, a two-sided type I error probability (α) of 0.05, a type II error probability (β) of 0.20 (corresponding to 80% power), a penalty λ of 2, and a conventional boundary Z value of 1.96 (equivalent to *P* = 0.05). The cumulative Z-curve was inspected to determine whether it crossed the conventional or TSA-adjusted monitoring boundaries, thereby evaluating the potential impact of random error on the meta-analytic findings.

(5) For adverse events, in addition to reporting the *RR*, the absolute event rates were reported for both groups (expressed as percentages and raw numbers/total numbers) to provide a more intuitive clinical interpretation.

### 2.7 GRADE evidence quality assessment

Two reviewers independently assessed the certainty of evidence for each outcome using GRADEpro GDT^1^ in accordance with the GRADE Handbook.^2^ Because all included studies were randomized controlled trials, the evidence began at high certainty and was subsequently rated down based on five domains: risk of bias, inconsistency, indirectness, imprecision, and publication bias. The final certainty ratings were classified as high, moderate, low, or very low. Discrepancies between the two reviewers were resolved through discussion; if consensus could not be reached, a third reviewer adjudicated the final rating.

## 3 Results

### 3.1 Literature screening process

**FIGURE 1:**
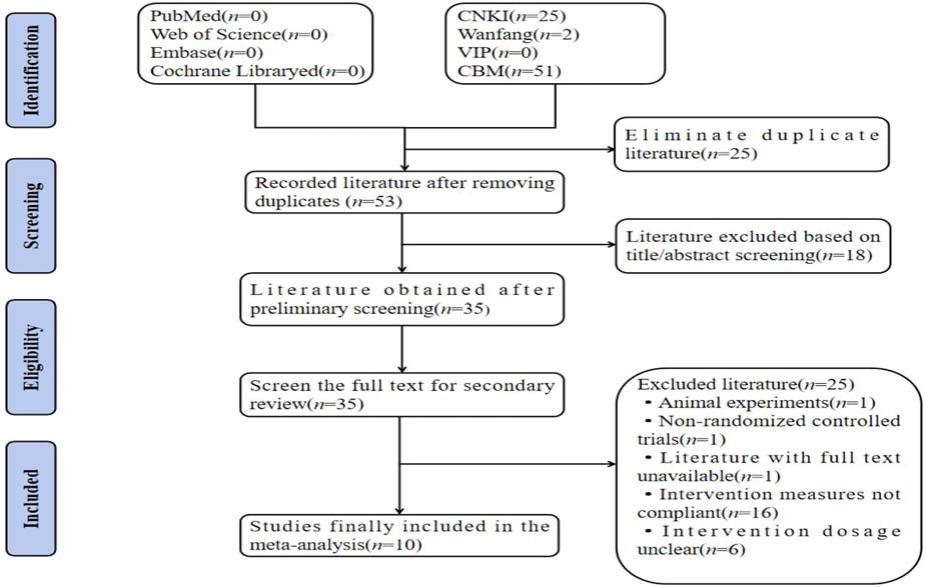
Study selection flowchart (PRISMA 2020).

A total of 78 articles were identified through database searches. Using Note Express, the articles were screened step by step according to the predefined inclusion and exclusion criteria. Ultimately, 10 studies (18–27) were included in this meta-analysis. All included studies were published in Chinese. The detailed screening process and the reasons for exclusion are presented in Figure 1.

### 3.2 Characteristics of included studies

Ten RCTs (18–27) involving 1,057 participants (529 in the intervention group and 528 in the control group) were included. The basic characteristics of the included studies are shown in Table 1. The weighted mean age of participants in each study, calculated using the formula recommended by the Cochrane Handbook, is presented in Supplementary Table S1.

**TABLE 1:** Baseline characteristics of included studies.

Study	Country	Sample size (*n*)		Age (years)		Disease duration		Intervention measures		treatment duration	Outcomes
		T	C	T	C	T	C	T	C		
Cai et al. (18)	China	41	40	49.5 ± 3.2	48.8 ± 3.6	-	-	KTC(6g/d, tid) + low-dose FMT (1 tablet/d, qd)	Low-dose FMT (1 tablet/d, qd)	3 months	➁➂➃➄➉
Meng 2024 (19)	China	40	40	48.71 ± 1.94	49.15 ± 2.57	10.44 ± 1.82 m	11.16 ± 1.42 m	KTC(6g/d, tid) + low-dose FMT (1 tablet/d, qd)	Low-dose FMT (1 tablet//d, qd)	3 months	➀➃➄⑪
Zhang 2024 (20)	China	81	81	47.45 ± 4.21	47.1 ± 3.97	2.36 ± 0.54 m	2.47 ± 0.60 m	KTC(6g/d, tid) + low-dose FMT (1 tablet/d, qd)	Low-dose FMT (1 tablet/d, qd)	3 months	➀➂➃➄➉⑪
Ke et al. (21)	China	47	47	48.1 ± 4.8	47.9 ± 4.6	13.1 ± 2.2 m	12.8 ± 2.4 m	KTC(6g/d, tid) + low-dose FMT (1 tablet/d, qd)	Low-dose FMT (1 tablet/d, qd)	60 days	➂➃➅➆➇➈⑪
Suo et al. (22)	China	49	49	51.32 ± 2.17	51.27 ± 2.19	3.17 ± 1.09 y	3.18 ± 1.08 y	KTC(6g/d, tid) + low-dose FMT (1 tablet/d, qd)	Low-dose FMT (1 tablet/d, qd)	3 months	➂➃➄➅➆➇➈
Gao et al. (23)	China	50	50	45.46 ± 2.33	45.54 ± 2.31	1.72 ± 1.12 y	1.95 ± 1.19 y	KTC(6g/d, tid) + low-dose FMT (1 tablet/d, qd)	Low-dose FMT (1 tablet/d, qd)	3 months	➀➁➂➃➄
Lu (24)	China	35	35	51.27 ± 3.31	51.35 ± 3.42	3.63 ± 1.08 y	3.48 ± 1.15 y	KTC(6g/d, tid) + low-dose FMT (1 tablet/d, qd)	Low-dose FMT (1 tablet/d, qd)	4 weeks	➀➂➃➄➅➆➇➈⑪
Guo (25)	China	71	71	52.26 ± 3.24	53.45 ± 3.36	-	-	KTC(6g/d, tid) + low-dose FMT (1 tablet/d, qd)	Low-dose FMT (1 tablet/d, qd)	2 months	➀➁➃➄➉⑪
Fu (26)	China	73	73	47.98 ± 4.92	48.41 ± 4.77	1.24 ± 0.62 m	1.27 ± 0.65 m	KTC(6g/d, tid) + low-dose FMT (1 tablet/d, qd)	Low-dose FMT (1 tablet/d, qd)	3 months	➀➁➂➃➄⑪
Wu et al. (27)	China	42	42	51.84 ± 3.54	52.03 ± 3.73	1.34 ± 0.38 y	1.41 ± 0.47 y	KTC(6g/d, tid) + low-dose FMT (1 tablet/d, qd)	Low-dose FMT (1 tablet/d, qd)	3 months	➀➂➃➄

T, treatment group; C, control group; “-”, Not reported; y, years; m, months; FMT, Femoston; KTC, Kuntai capsule. ➀ Clinical response rate; ➁ Kupperman score; ➂E_2_; ➃ FSH; ➄LH; ➅TC; ➆TG; ➇LDL-C; ➈ HDL-C; ➉ Endometrial thickness; ⑪ Adverse events.

### 3.3 Risk of bias assessment

All included studies were RCTs. Eight studies (19–23, 25–27) used a random number table for group allocation and were rated as “low risk;” the other two studies (18, 24) only stated that participants were “randomly assigned” without further detail and were thus rated as “unclear risk.” Allocation concealment was not reported in any of the included studies. Hence, the risk was assessed as “unclear risk.” Blinding (double- or triple-blind) was not reported in any of the included studies. The risk was evaluated as “unclear risk.” There were no missing outcome data in any of the studies. The outcome data were complete and were rated as “low risk.” All expected outcomes were reported in all included studies. The risk of selective reporting bias was considered “low risk.” None of the included studies had other potential sources of bias. The risk was evaluated as “low risk.”

**FIGURE 2:**
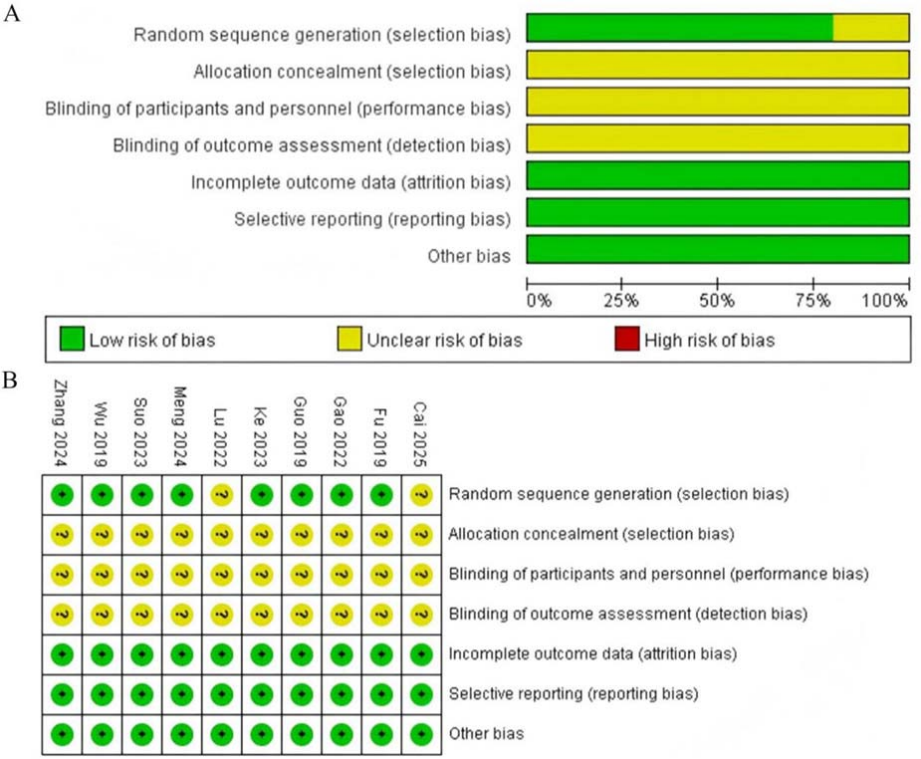
Risk of bias assessment chart. **(A)** Risk of bias graph: summary of the risk of bias across all included studies, presented as percentages. **(B)** Risk of bias summary: assessment of each individual study. 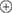 : low risk of bias; 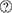 : unclear risk of bias.

In summary, methodological deficiencies in allocation concealment and blinding across the included studies resulted in low overall methodological quality, which may introduce a potential risk of bias (see Figure 2).

### 3.4 Results of meta-analysis

#### 3.4.1 Kupperman score

**FIGURE 3:**
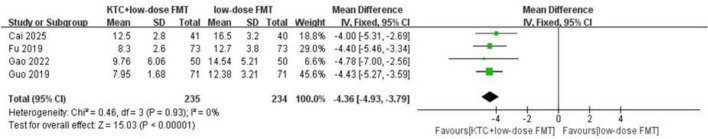
Forest plot comparing Kupperman scores between KTC combined with low-dose FMT and low-dose FMT alone in patients with PMS.

Four studies (18, 23, 25, 26) involving 469 participants reported Kupperman scores for both groups. Heterogeneity was low (*I*^2^ = 0%, *P* = 0.93); therefore, a fixed-effects model was applied. Meta-analysis revealed a statistically significant between-group difference [*MD* = −4.36, 95% CI (−4.93, −3.79), *P* < 0.00001], indicating that KTC combined with low-dose FMT had a significant advantage in improving Kupperman scores compared with low-dose FMT alone in patients with PMS (Figure 3).

#### 3.4.2 E_2_ levels

**FIGURE 4:**
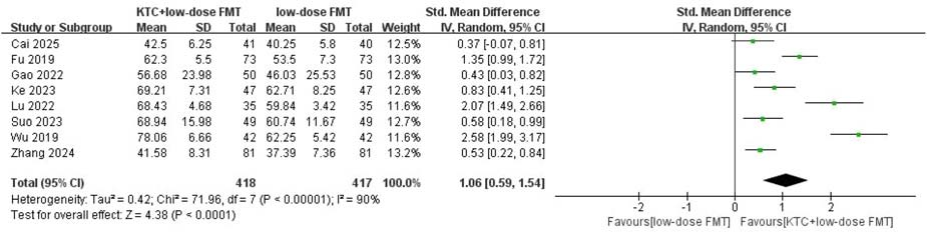
Forest plot comparing E_2_ levels between KTC combined with low-dose FMT and low-dose FMT alone in patients with PMS.

Eight studies (18, 20–24, 26, 27) involving 835 participants reported E_2_ levels for both groups. Substantial between-study heterogeneity was noted (*I*^2^ = 90%, *P* < 0.00001); therefore, a random-effects model was applied. The results showed a statistically significant difference between the two groups (*SMD* = 1.06, 95% CI [0.59, 1.54], *P* < 0.0001), indicating that KTC combined with low-dose FMT significantly increased E_2_ levels in patients with PMS (Figure 4).

**FIGURE 5:**
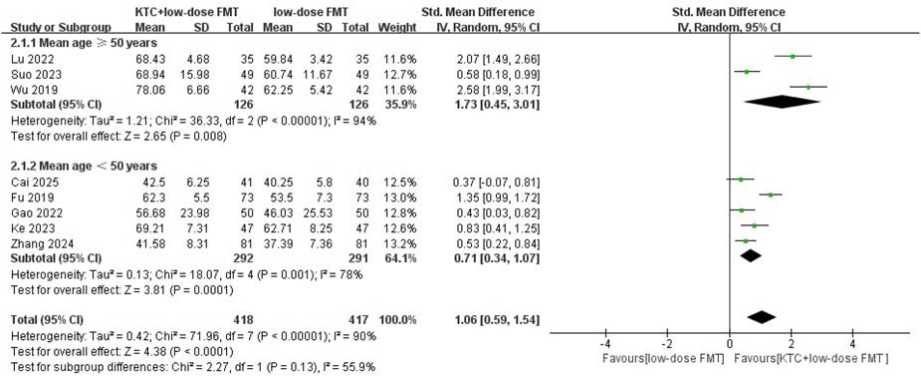
Forest plot of subgroup analysis for E_2_ levels by mean age.

**FIGURE 6:**
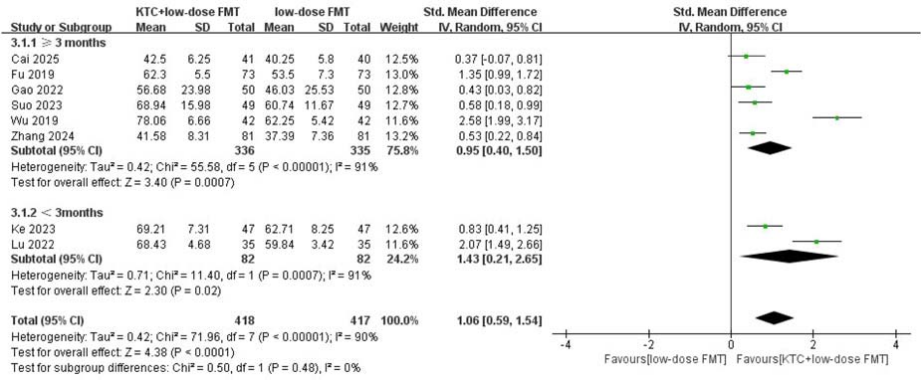
Forest plot of subgroup analysis for E_2_ levels by treatment duration.

Given the substantial heterogeneity observed for the outcome, prespecified subgroup analyses were performed by mean age and treatment duration. The between-subgroup differences were not statistically significant for the age subgroup (χ^2^ = 2.27, df = 1, *P* for interaction = 0.13, *I*^2^ = 55.9%) or the treatment duration subgroup (χ^2^ = 0.50, df = 1, *P* for interaction = 0.48, *I*^2^ = 0%) (see Figures 5, 6).

#### 3.4.3 FSH levels

**FIGURE 7:**
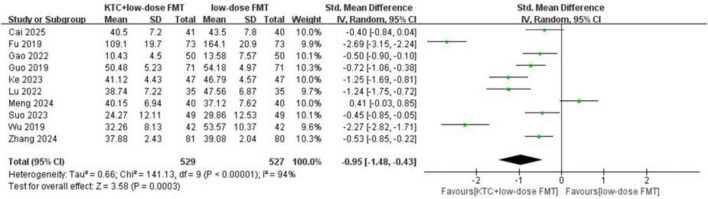
Forest plot comparing FSH levels between KTC combined with low-dose FMT and low-dose FMT alone in patients with PMS.

Ten studies (18–27) involving 1,057 participants reported FSH levels for both groups. Heterogeneity was substantial (*I*^2^ = 94%, *P* < 0.00001); a random-effects model was applied. The results showed a statistically significant difference between the two groups [*SMD* = −0.95, 95% CI (−1.48, −0.43), *P* = 0.0003], indicating that KTC combined with low-dose FMT significantly reduced FSH levels compared with low-dose FMT alone in patients with PMS (Figure 7).

**FIGURE 8:**
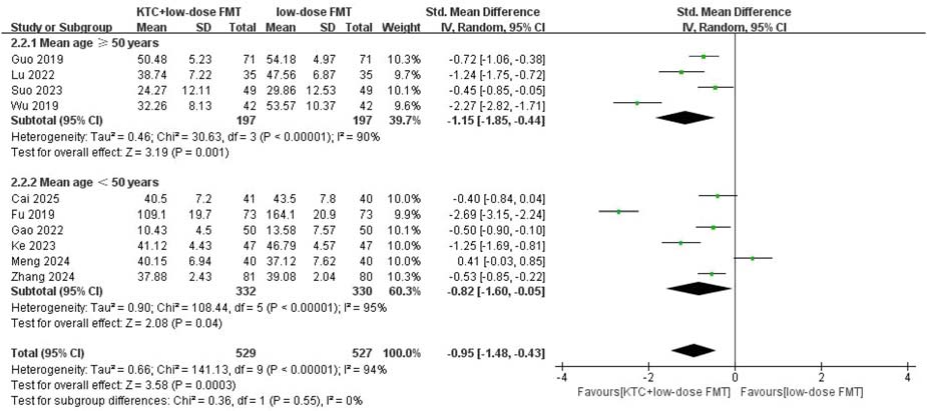
Forest plot of subgroup analysis for FSH levels by mean age.

**FIGURE 9:**
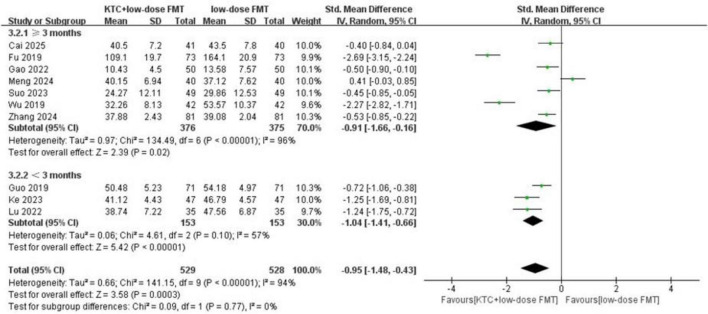
Forest plot of subgroup analysis for FSH levels by treatment duration.

Given the substantial heterogeneity of the outcome, prespecified subgroup analyses were performed by mean age and treatment duration. The between-subgroup differences were not statistically significant for the age subgroup (χ^2^ = 0.36, df = 1, *P* for interaction = 0.55, *I*^2^ = 0%) or the treatment duration subgroup (χ^2^ = 0.09, df = 1, *P* for interaction = 0.77, *I*^2^ = 0%) (see Figures 8, 9).

#### 3.4.4 LH levels

**FIGURE 10:**
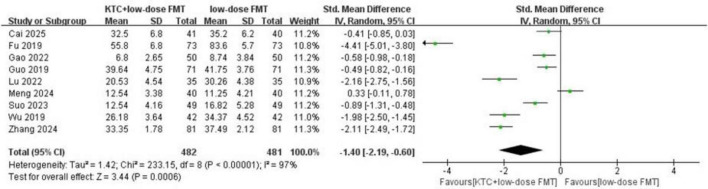
Forest plot comparing LH levels between KTC combined with low-dose FMT and low-dose FMT alone in patients with PMS.

Nine studies (18–20, 22–27) involving 963 participants reported LH levels for both groups. Heterogeneity was substantial (*I*^2^ = 97%, *P* < 0.00001); a random-effects model was applied. The results showed a statistically significant difference between the two groups [*SMD* = −1.40, 95% CI (−2.19, −0.60), *P* = 0.0006], indicating that KTC combined with low-dose FMT significantly reduced LH levels compared with low-dose FMT alone in patients with PMS (Figure 10).

**FIGURE 11:**
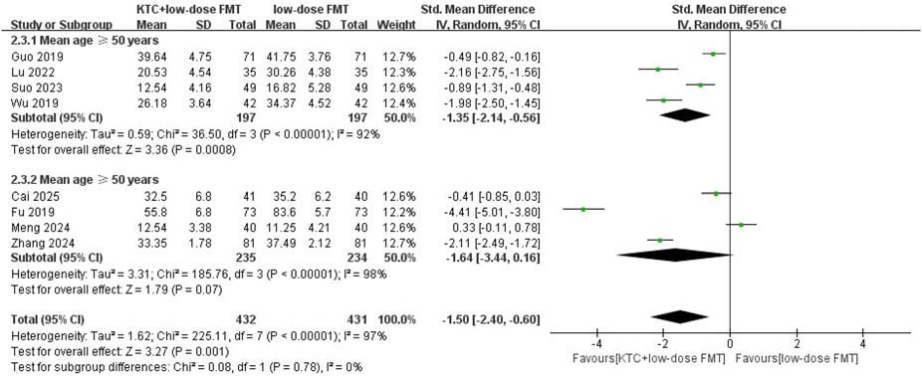
Forest plot of subgroup analysis for LH levels by mean age.

**FIGURE 12:**
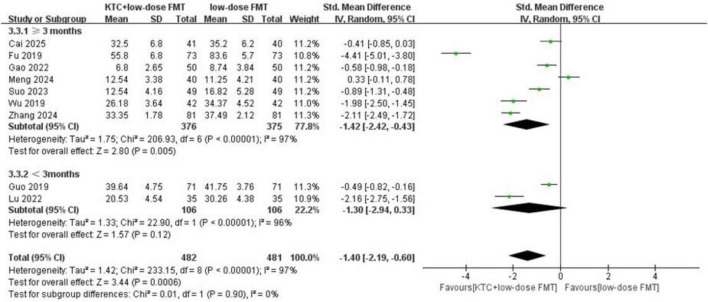
Forest plot of subgroup analysis for LH levels by treatment duration.

Given the substantial heterogeneity of the outcome, prespecified subgroup analyses were performed by mean age and treatment duration. The between-subgroup differences were not statistically significant for the age subgroup (χ^2^ = 0.08, df = 1, *P* for interaction = 0.78, *I*^2^ = 0%) or the treatment duration subgroup (χ^2^ = 0.01, df = 1, *P* for interaction = 0.90, *I*^2^ = 0%) (see Figures 11, 12).

#### 3.4.5 TC levels

**FIGURE 13:**
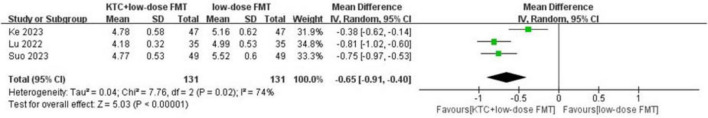
Forest plot comparing TC levels between KTC combined with low-dose FMT and low-dose FMT alone in patients with PMS.

Three studies (21, 22, 24) involving 262 participants reported TC levels for both groups. Heterogeneity was substantial (*I*^2^ = 74%, *P* = 0.02); a random-effects model was applied. The results showed a statistically significant difference between the two groups [*MD* = −0.65, 95% CI (−0.91, −0.40), *P* < 0.00001], indicating that KTC combined with low-dose FMT significantly reduced TC levels compared with low-dose FMT alone in patients with PMS (Figure 13).

**FIGURE 14:**
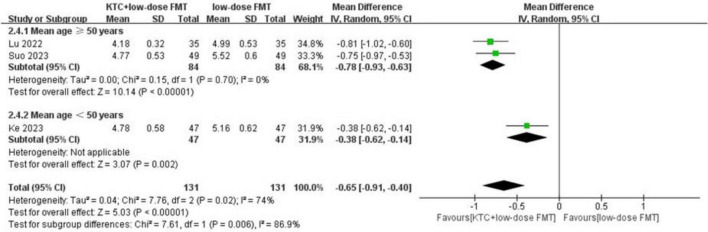
Forest plot of subgroup analysis for TC levels by mean age.

**FIGURE 15:**
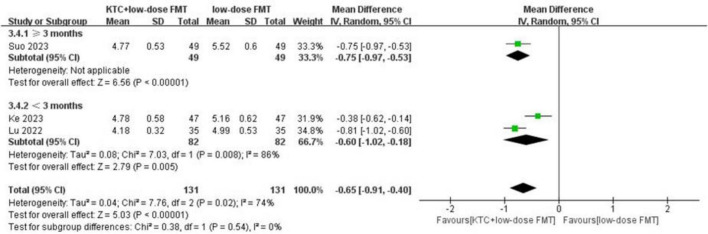
Forest plot of subgroup analysis for TC levels by treatment duration.

Given that *I*^2^ ≥ 50%, prespecified subgroup analyses were performed (see Figures 14, 15). Subgroup analysis stratified by mean age revealed a statistically significant interaction (χ^2^ = 7.61, df = 1, *P* for interaction = 0.006, *I*^2^ = 86.9%). The reduction in TC levels appeared to be greater in patients mean aged ≥ 50 years (*MD* = −0.78, 95% CI [−0.93, −0.63]) than in those mean aged < 50 years [*MD* = −0.38, 95% CI (−0.62, −0.14)]. However, the number of studies in the subgroups was small, and the confidence intervals were wide; therefore, this finding should be interpreted with caution. Subgroup analysis stratified by treatment duration showed no statistically significant between-subgroup difference (χ^2^ = 0.38, df = 1, *P* for interaction = 0.54, *I*^2^ = 0%).

#### 3.4.6 TG levels

**FIGURE 16:**
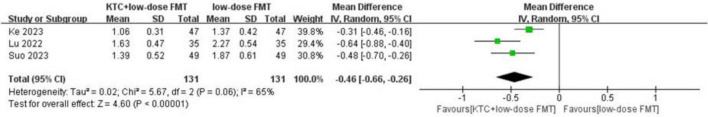
Forest plot comparing TG levels between KTC combined with low-dose FMT and low-dose FMT alone in patients with PMS.

Three studies (21, 22, 24) involving 262 participants reported TG levels for both groups. Heterogeneity was moderate (*I*^2^ = 65%, *P* = 0.06); a random-effects model was applied. The results showed a statistically significant difference between the two groups (*MD* = −0.46, 95% CI [−0.66, −0.26], *P* < 0.00001), indicating that KTC combined with low-dose FMT significantly reduced TG levels compared with low-dose FMT alone in patients with PMS (Figure 16).

**FIGURE 17:**
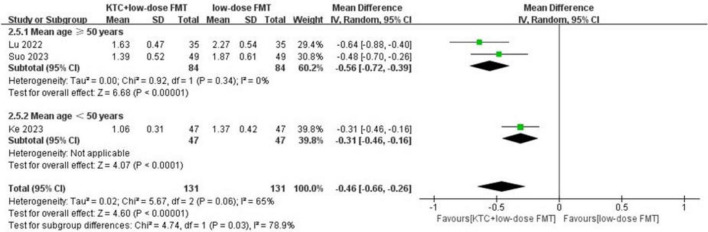
Forest plot of subgroup analysis for TG levels by mean age.

**FIGURE 18:**
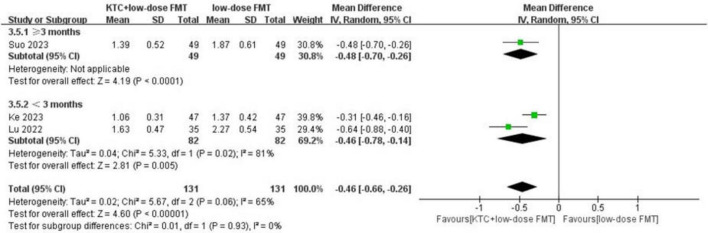
Forest plot of subgroup analysis for TG levels by treatment duration.

Given that *I*^2^ ≥ 50%, prespecified subgroup analyses were performed (see Figures 17, 18). Subgroup analysis stratified by mean age revealed a statistically significant interaction (χ^2^ = 4.74, df = 1, *P* for interaction = 0.03, *I*^2^ = 78.9%). The reduction in TG levels appeared to be greater in patients mean aged ≥ 50 years [*MD* = −0.56, 95% CI (−0.72, −0.39)] than in those mean aged < 50 years [*MD* = −0.31, 95% CI (−0.46, −0.16)]. However, the number of studies in the subgroups was small, and the confidence intervals were wide; therefore, this finding should be interpreted with caution. Subgroup analysis stratified by treatment duration showed no statistically significant between-subgroup difference (χ^2^ = 0.01, df = 1, *P* for interaction = 0.93, *I*^2^ = 0%).

#### 3.4.7 LDL-C levels

**FIGURE 19:**
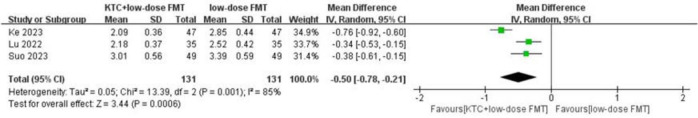
Forest plot comparing LDL-C levels between KTC combined with low-dose FMT and low-dose FMT alone in patients with PMS.

Three studies (21, 22, 24) involving 262 participants reported LDL-C levels for both groups. Heterogeneity was substantial (*I*^2^ = 85%, *P* = 0.001); a random-effects model was applied. The results showed a statistically significant difference between the two groups [*MD* = −0.50, 95% CI (−0.78, −0.21), *P* = 0.0006], indicating that KTC combined with low-dose FMT significantly reduced LDL-C levels compared with low-dose FMT alone in patients with PMS (Figure 19).

**FIGURE 20:**
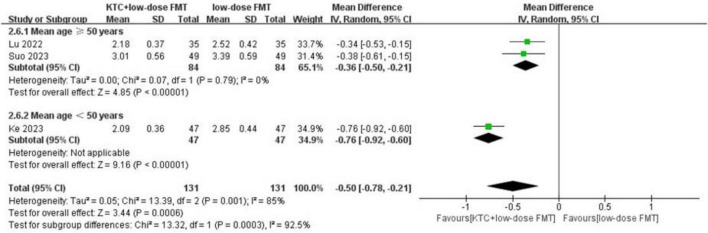
Forest plot of subgroup analysis for LDL-C levels by mean age.

**FIGURE 21:**
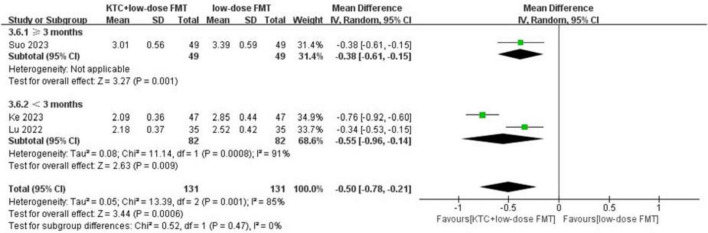
Forest plot of subgroup analysis for LDL-C levels by treatment duration.

Given that *I*^2^ ≥ 50%, prespecified subgroup analyses were performed (see Figures 20, 21). Subgroup analysis stratified by mean age revealed a statistically significant interaction (χ^2^ = 13.32, df = 1, *P* for interaction = 0.0003, *I*^2^ = 92.5%). The reduction in LDL-C levels appeared to be greater in patients mean aged < 50 years [*MD* = −0.76, 95% CI (−0.92, −0.60)] than in those mean aged ≥ 50 years [*MD* = −0.36, 95% CI (−0.50, −0.21)]. However, the number of studies in the subgroups was small, and the confidence intervals were wide; therefore, this finding should be interpreted with caution. Subgroup analysis stratified by treatment duration showed no statistically significant between-subgroup difference (χ^2^ = 0.52, df = 1, *P* for interaction = 0.47, *I*^2^ = 0%).

#### 3.4.8 HDL-C levels

**FIGURE 22:**
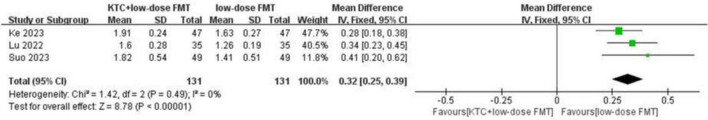
Forest plot comparing HDL-C levels between KTC combined with low-dose FMT and low-dose FMT alone in patients with PMS.

Three studies (21, 22, 24) reported HDL-C levels for both groups, involving a total of 262 participants. Heterogeneity was low (*I*^2^ = 0%, *P* = 0.49); a fixed-effects model was applied. The results showed a statistically significant difference between the two groups [*MD* = 0.32, 95% CI (0.25, 0.39), *P* < 0.00001], indicating that KTC combined with low-dose FMT was superior to low-dose FMT alone in improving HDL-C levels in patients with PMS (Figure 22).

#### 3.4.9 Endometrial thickness

**FIGURE 23:**
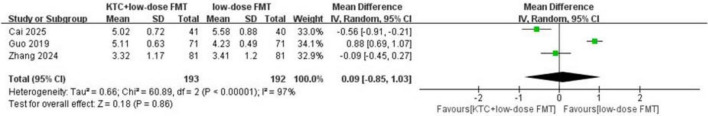
Forest plot comparing endometrial thickness between KTC combined with low-dose FMT and low-dose FMT alone in patients with PMS.

Three studies (18, 20, 25) involving 385 participants reported endometrial thickness for both groups. Heterogeneity was substantial (*I*^2^ = 97%, *P* < 0.00001); a random-effects model was applied. The results showed no statistically significant difference between the two groups [*MD* = 0.09, 95% CI (−0.85, 1.03), *P* = 0.86] (Figure 23).

**FIGURE 24:**
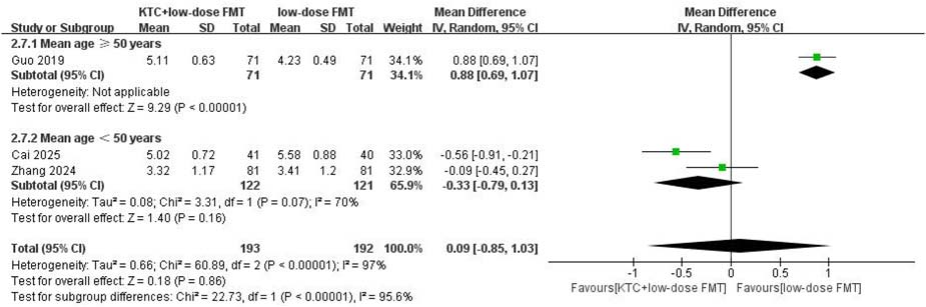
Forest plot of subgroup analysis for endometrial thickness by mean age.

**FIGURE 25:**
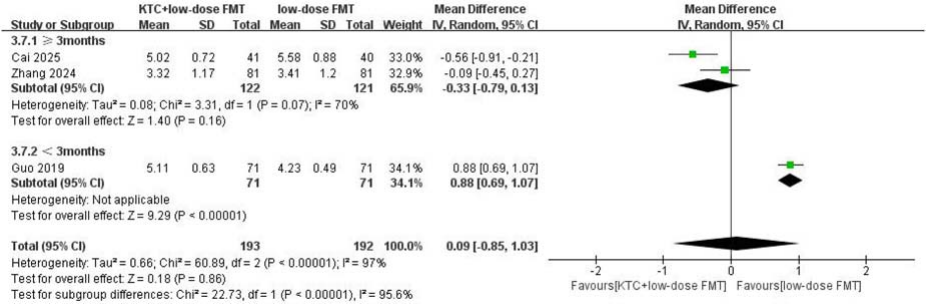
Forest plot of subgroup analysis for endometrial thickness by treatment duration.

Given that *I*^2^ ≥ 50%, prespecified subgroup analyses were performed (see Figures 24, 25). Subgroup analysis stratified by mean age revealed a statistically significant interaction (χ^2^ = 22.73, df = 1, *P* for interaction < 0.00001, *I*^2^ = 95.6%). A decreasing trend in endometrial thickness was observed in patients mean aged < 50 years [*MD* = −0.33, 95% CI (−0.79, 0.13)] compared with those mean aged ≥ 50 years [*MD* = 0.88, 95% CI (0.69, 1.07)]. Subgroup analysis stratified by treatment duration also showed a statistically significant interaction (χ^2^ = 22.73, df = 1, *P* for interaction < 0.00001, *I*^2^ = 95.6%). Compared with the < 3 months group [*MD* = 0.88, 95% CI (0.69, 1.07)], the ≥ 3 months group [*MD* = −0.33, 95% CI (−0.79, 0.13)] exhibited a decreasing trend in endometrial thickness (Supplementary Table 3). However, the number of studies in the subgroups was small, and the confidence intervals were wide; therefore, these findings should be interpreted with caution.

#### 3.4.10 Clinical response rate

**FIGURE 26:**
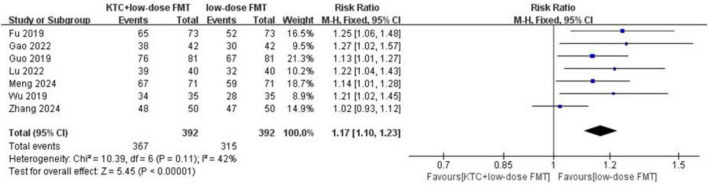
Forest plot comparing clinical response rate KTC combined with low-dose FMT and low-dose FMT alone in patients with PMS.

Seven studies (19, 20, 23–27) involving 784 participants reported the clinical response rate for both groups. Heterogeneity test revealed *I*^2^ = 42%, *P* = 0.11; a fixed-effects model was applied. Meta-analysis showed a statistically significant between-group difference [*RR* = 1.17, 95% CI (1.10, 1.23), *P* < 0.00001], indicating that KTC combined with low-dose FMT significantly improved the clinical response rate compared with low-dose FMT alone in patients with PMS (Figure 26).

#### 3.4.11 Adverse events

**FIGURE 27:**
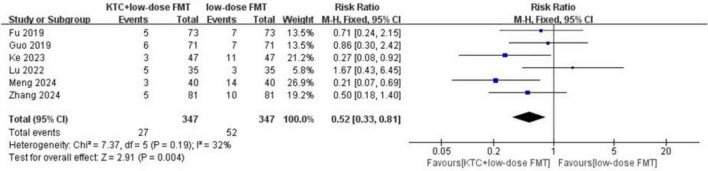
Forest plot comparing adverse events rate KTC combined with low-dose FMT and low-dose FMT alone in patients with PMS.

Among the 10 included studies, six studies (19–21, 24–26) reported the occurrence of adverse events in both groups, involving 694 participants. Heterogeneity was low (*I*^2^ = 32%, *P* = 0.19); a fixed-effects model was applied. The results showed a statistically significant difference between the two groups [*RR* = 0.52, 95% CI (0.33, 0.81), *P* = 0.004], indicating that the combination therapy reduced the incidence of adverse events compared with low-dose FMT alone (Figure 27). The absolute event rate was 7.8% (27/347) in the combination therapy group and 15.0% (52/347) in the control group.

In addition, the incidence of specific adverse events was analyzed. Compared with the control group, the combination therapy significantly reduced the incidence of breast tenderness, whereas no statistically significant differences were observed in the incidence of vaginal bleeding, nausea, abdominal pain, or headache (all *P* > 0.05) (Table 2). Other adverse events (fatigue, gastrointestinal discomfort, weight gain, and vaginal pruritus) were reported in fewer than two studies and were not subjected to meta-analysis; their distributions are presented in Table 3.

**TABLE 2:** Meta-analysis results of selected adverse events.

Adverse event	No. of studies	T	C	Heterogeneity (*P*)	*I* ^2^	Model	*RR* (95% CI)	*P*-value
		Events/ Total	Events/ Total					
Breast tenderness	6	8/347	19/347	0.58	0%	Fixed	0.44(0.20, 0.96)	0.04
Nausea	5	8/312	11/312	0.63	0%	Fixed	0.74 (0.31, 1.77)	0.50
Vaginal bleeding	3	1/122	3/122	0.44	0%	Fixed	0.56 (0.12, 2.57)	0.45
Abdominal pain	2	3/87	4/87	0.28	15%	Fixed	0.75 (0.17, 3.27)	0.70
Headache	2	3/106	5/106	0.85	0%	Fixed	0.60 (0.15, 2.42)	0.47

T, Treatment group; C, Control group.

**TABLE 3:** Distribution of adverse events in the two groups across included studies (number of cases).

Study	Adverse events	
	T	C
Meng (19)	Abdominal pain (2 cases), Nausea (1 case), vaginal bleeding (1 case), Breast tenderness (1 case)	Abdominal pain (1 case), Nausea (1 case), Breast tenderness (1 case)
Zhang (20)	Nausea (2 cases), Breast tenderness (4 cases)	Nausea (3 cases), Breast tenderness (4 cases)
Ke et al. (21)	Nausea (1 case), Abdominal pain (1 case), Breast tenderness (1 case)	Nausea (4 cases), Abdominal pain (3 cases), Breast tenderness (2 cases), Vaginal bleeding (2 cases)
Lu (24)	Headache (2 cases), Fatigue (2 cases), Breast tenderness (1 case)	Headache (3 cases), Fatigue (4 cases), Vaginal bleeding (1 case), Breast tenderness (2 cases)
Guo (25)	Headache (1 case), Nausea (2 cases)	Headache (2 cases), Weight gain (3 cases), Breast tenderness (8 cases), Vaginal itching (1 case)
Fu (26)	Gastric discomfort (2 cases), Breast tenderness (1 case), Nausea (2 cases)	Gastric discomfort (2 cases), Breast tenderness (2 cases), Nausea (3 cases)

T, treatment group; C, control group.

#### 3.4.12 Sensitivity analysis

Sensitivity analyses were performed for outcomes with substantial heterogeneity (*I*^2^ ≥ 50%) to assess the robustness of pooled estimates (Supplementary Figure 1; Supplementary Tables 2–8).

1. Leave-one-out sensitivity analysis showed that the direction and statistical significance of the pooled effect sizes for E_2_, LH, FSH, and endometrial thickness did not change substantially, indicating that the combined results were robust.

2. Lipid parameters

(1) The study by Ke (21) was identified as the primary source of heterogeneity for TC and LDL-C. After exclusion of this study, heterogeneity was substantially reduced for TC (*I*^2^ = 0%, *P* = 0.70), with a pooled effect estimate of *MD* = −0.78 [95% CI (−0.93, −0.63), *P* < 0.00001]. Similarly, heterogeneity was eliminated for LDL-C (*I*^2^ = 0%, *P* = 0.79), yielding a pooled estimate of *MD* = −0.36 [95% CI (−0.50, −0.21), *P* < 0.01]. The conclusions for both TC and LDL-C remained consistent with those before exclusion, demonstrating the robustness of the pooled results.

(2) After excluding Lu (24), heterogeneity for TG decreased to *I*^2^ = 35% (*P* = 0.22), with a pooled *MD* of −0.37 [95% CI (−0.53, −0.21), *P* < 0.01]. Following exclusion of Ke (21), heterogeneity decreased to *I*^2^ = 0% (*P* = 0.34), yielding a pooled *MD* of −0.56 [95% CI (−0.72, −0.39), *P* < 0.01]. The conclusions remained consistent with those before exclusion, further demonstrating the robustness of the combined results.

#### 3.4.13 Publication bias assessment

**FIGURE 28:**
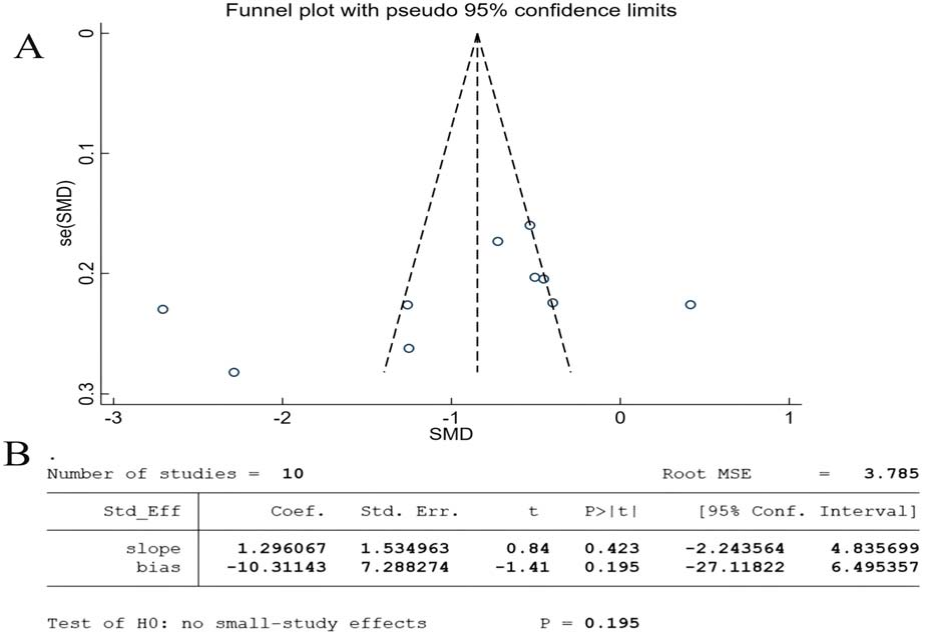
Assessment of publication bias for FSH levels. **(A)** Funnel plot of FSH levels for the comparison of KTC combined with low-dose FMT versus low-dose Femoston alone. The horizontal axis shows the *SMD*, and the vertical axis shows the standard error (SE). Each circle represents an individual study. **(B)** Egger’s regression test (*P* = 0.195), indicating no significant publication bias.

According to the Cochrane Handbook, publication bias was formally assessed only for FSH. As shown in Figure 28A, most studies were concentrated on the right side of the funnel plot, with only two small-sample studies located on the lower left side, and three studies fell outside the 95% confidence limits, indicating a mild asymmetry. To reduce subjectivity in the visual interpretation of the funnel plot, Egger’s test was further performed, which yielded a *P* value of 0.195 (*P* > 0.05), suggesting no significant publication bias among the included studies (Figure 28B).

#### 3.4.14 Trial sequential analysis

**FIGURE 29:**
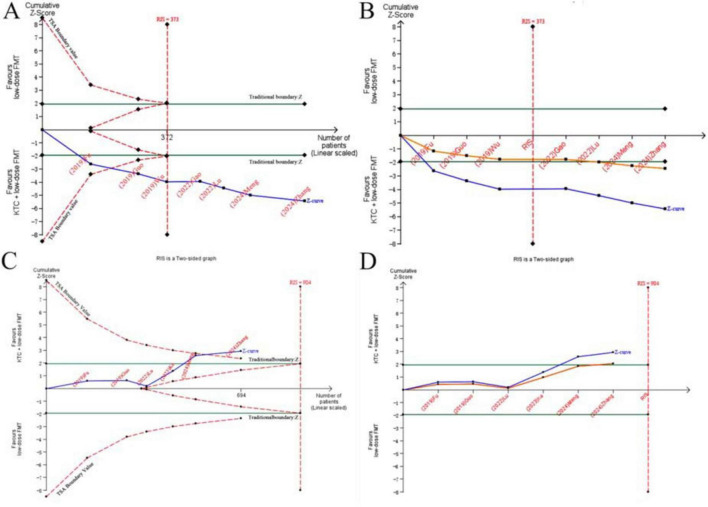
Trial sequential analysis (TSA). **(A)** TSA of the clinical response rate. **(B)** Penalized statistical assessment of the clinical response rate. **(C)** TSA of the adverse events rate. **(D)** Penalized statistical assessment of adverse events rate.

Clinical response rate: Seven trials reported the clinical response rate for PMS. The control group event rate was set at 80%, the intervention group event rate at 93.6%, and the relative risk reduction at −17%. TSA demonstrated that the cumulative Z-curve (blue solid line) crossed the conventional boundary (green solid line, *Z* = 1.96) after inclusion of the first trial (26); crossed the trial sequential monitoring boundary (red dashed line) after inclusion of the second trial (25); and exceeded the required information size (RIS = 373) after inclusion of the fourth trial (27) (Figure 29A). These findings indicate that combination therapy significantly improves the clinical response rate and that the meta-analysis result is robust against random error. To further assess the reliability of this conclusion (Figure 29B). The penalized Z-curve (orange solid line) crossed the conventional boundary after inclusion of the sixth trial (19), corroborating the robustness of the finding.

Adverse events: Based on the pooled adverse event rates, the control group event rate was set at 14.9%, the intervention group event rate at 7.7%, and the relative risk reduction at 48.3%. TSA showed that the cumulative Z-curve (blue solid line) crossed the conventional boundary after inclusion of the fifth trial (19) and crossed the trial sequential monitoring boundary (red dashed line) after inclusion of the sixth trial (20) (Figure 29C). Although the cumulative sample size did not reach the required information size (RIS = 904), a conclusive positive result was established before this threshold was attained, suggesting that the finding is reliable. The penalized Z-curve analysis (orange solid line) confirmed this conclusion, with the curve crossing the conventional boundary after inclusion of the sixth trial (20) (Figure 29D).

#### 3.4.15 GRADE quality of evidence assessment

The certainty of evidence for all outcomes was evaluated using GRADEpro GDT. The certainty was rated as moderate for the clinical response rate, Kupperman score, and HDL-C levels; low for TC, TG, and LDL-C levels; and very low for E_2_, LH, endometrial thickness, and adverse events (see Table 4).

**TABLE 4:** GRADE certainty of evidence and reasons for downgrading.

Outcome measures	No. of studies	Certainty assessment					No. of patients		Effect size (95%CI)	Quality grade	
		Risk of bias	Inconsistency	Indirectness	Imprecision	Other considerations	KTC + low-dose FMT	Low-dose FMT			
Clinical response rate	7	Serious^a^	Not serious	Not serious	Not serious	None	367/392 (93.6%)	315/392 (80.4%)	*RR* = 1.17 (1.10, 1.23)	⊕⊕⊕○	Moderate^a^
Kupperman score	4	Serious^a^	Not serious	Not serious	Not serious	None	235	234	*MD* = −4.36 (−4.93, −3.79)	⊕⊕⊕○	Moderate^a^
E_2_	8	Serious^a^	Serious^b^	Not serious	Serious^c^	None	418	417	*SMD* = 1.06 (0.59, 1.54)	⊕○○○	Very low^a,b,c^
LH	9	Serious^a^	Serious^b^	Not serious	Serious^c^	None	482	481	*SMD* = −1.40 (−2.19, −0.60)	⊕○○○	Very lowa^,b,c^
FSH	10	Serious^a^	Serious^b^	Not serious	Serious^c^	None	529	528	*SMD* = −0.95 (−1.48, −0.43)	⊕○○○	Very low^a,b,c^
Endometrial thickness	3	Serious^a^	Very serious^d^	Not serious	Serious^e^	None	193	192	*MD* = 0.09 (−0.85, 1.03)	⊕○○○	Very low^a,d,e^
TC	3	Serious^a^	Not serious	Not serious	Serious^e^	None	131	131	*MD* = −0.65 (−0.91, −0.40)	⊕⊕○○	Low^a,e^
TG	3	Serious^a^	Not serious	Not serious	Serious^e^	None	131	131	*MD* = −0.46 (−0.66, −0.26)	⊕⊕○○	Low^a,e^
LDL-C	3	Serious^a^	Not serious	Not serious	Serious^e^	None	131	131	*MD* = −0.50 (−0.78, −0.21)	⊕⊕○○	Low^a,e^
HDL-C	3	Serious^a^	Not serious	Not serious	Not serious	None	131	131	*MD* = 0.32 (0.25, 0.39)	⊕⊕⊕○	Moderate^a^
Adverse events	6	Serious^a^	Serious^f^	Not serious	Serious^c^	None	27/347 (7.8%)	52/347 (15.0%)	*RR* = 0.52 (0.33, 0.81)	⊕○○○	Very low^a,c,f^

*^a^*There was a lack of clear reporting on allocation concealment and blinding.

*^b^*Unexplained heterogeneity.

*^c^*Wide 95% confidence interval.

*^d^*The overall effect estimate exceeded the threshold of no effect, and the Guo (25) study indicated the opposite.

*^e^*Small sample size with a wide 95% confidence interval.

*^f^*Lu (24) study pointed in the opposite direction. The heterogeneity for TC, TG, and LDL-C was explained by sensitivity analysis; therefore, the evidence was not downgraded for inconsistency.

## 4 Discussion

### 4.1 Main findings

This systematic review evaluated the efficacy and safety of KTC combined with low-dose FMT in the treatment of PMS. Based on the available evidence, compared with low-dose FMT alone, the combination therapy showed potential advantages in alleviating PMS (reducing Kupperman scores), regulating sex hormone and lipid levels, and improving medication safety. However, the GRADE assessment indicated that the certainty of evidence for most outcomes was low to very low, and substantial heterogeneity persisted across several outcomes and was not fully accounted for by prespecified subgroup analyses. Therefore, the results of this study should be interpreted with caution.

### 4.2 Comparison with previous studies

In 2021, Wang et al. (28) conducted a systematic review on TCM combined with FMT for the treatment of PMS, and reported that TCM combined with FMT was superior to FMT alone in terms of clinical efficacy, improvement of sex hormone levels, and reduction of adverse events. However, several limitations should be noted. First, the types of TCM included were diverse (e.g., KTC, Wuji Baifeng Pills, Kunning’an Pills, and self-formulated TCM decoctions), and the study did not focus on a specific compound preparation nor did it restrict the dosage of FMT. The pooled analysis may have masked the true effects of individual drugs. Second, the study only reported the overall incidence of adverse events without categorizing or analyzing specific types of adverse events, making it impossible to determine the impact of the combination therapy on particular adverse events.

In light of these limitations, the present study was optimized in the following aspects: (1) We focused on a single TCM formulation (e.g., KTC) and strictly limited the dose of FMT to evaluate the clinical value of this specific combination regimen. (2) We systematically assessed the effects of KTC combined with low-dose FMT on the lipid profile (TC, TG, LDL-C, and HDL-C). (3) We performed meta-analyses of specific types of adverse events, thereby providing more refined evidence for clinical safety evaluation. (4) We applied GRADE and TSA to enhance the transparency and robustness of the conclusions from a methodological perspective.

### 4.3 Potential mechanisms of combination therapy for PMS

The core mechanism underlying PMS is declining ovarian reserve and the consequent reduction in endogenous E_2_ production. Diminished E_2_ attenuates negative feedback inhibition on the hypothalamus and pituitary, resulting in compensatory elevations in FSH and LH. This neuroendocrine imbalance constitutes a key mechanistic underpinning for the diverse symptomatology of PMS. Regarding vasomotor symptoms, reduced E_2_ levels lead to hyperactivation of estrogen-sensitive neurons within the hypothalamus, which promotes pulsatile gonadotropin-releasing hormone release and precipitates vasomotor dysfunction, manifesting as hot flashes and night sweats (29). With respect to neuropsychiatric symptoms, E_2_ deficiency can interfere with the serotonergic and γ-aminobutyric acid neurotransmission, compromising hippocampal and prefrontal neuroplasticity and neuroprotection, thereby increasing vulnerability to anxiety, depression, and sleep disturbances (30). In terms of metabolic and cardiovascular effects, the cardioprotective effects of estrogen are attenuated as levels decline, contributing to endothelial dysfunction, increased arterial stiffness, and dyslipidemia, which collectively elevate postmenopausal cardiovascular risk (31).

Clinically, HRT is employed to compensate for estrogen deficiency. FMT, a compound formulation of estradiol and dydrogesterone, restores negative feedback inhibition on the hypothalamus-pituitary axis by exogenously supplementing estrogen, suppressing the pulse amplitude of gonadotropin-releasing hormone, and reducing the secretion of FSH and LH, thereby alleviating vasomotor symptoms such as hot flashes (32). The dydrogesterone component protects the endometrium from unopposed estrogen-induced proliferation (32). Regarding lipid regulation, Stevenson et al. (33) found that both 1 mg and 2 mg estradiol combined with dydrogesterone improve dyslipidemia, with no significant difference in efficacy between the two doses; moreover, dydrogesterone has a relatively mild effect on lipid profiles and does not attenuate the beneficial effects of estrogen. However, FMT only supplies exogenous estrogen and cannot fully replicate the complex physiological milieu of a functioning ovary (34).

KTC is a compound TCM formulation composed of six herbs: *Rehmanniae Radix Praeparata* (Shu Dihuang), *Colla Corii Asini* (Ejiao), *Scutellariae Radix* (Huangqin), *Coptidis Rhizoma* (Huanglian), *Paeoniae Radix Alba* (Baishao), and *Poria* (Fuling). In TCM theory, this combination is characterized as “nourishing yin and clearing heat, calming the mind, alleviating vexation.” Existing studies have shown that KTC improves ovarian function by regulating the Hypothalamus-Pituitary-Ovarian axis (35). Furthermore, certain components of KTC—notably *Rehmanniae Radix Praeparata* and *Colla Corii Asini*—possess phytoestrogen-like properties and can selectively activate estrogen receptors ERα and ERβ, thereby compensating for insufficient endogenous estrogen levels. These mechanisms contribute to the alleviation of PMS and the improvement of estrogen-deficiency-associated dyslipidemia (9).

Based on the findings of this meta-analysis, the combination therapy showed superior clinical efficacy compared with low-dose FMT alone in ameliorating PMS, regulating sex hormone levels, and improving lipid profiles, suggesting a potential synergistic or complementary interaction between the two interventions. However, mechanistic studies specifically targeting this combination regimen are currently lacking, and high-quality mechanism-oriented clinical trials are warranted to further validate these findings.

### 4.4 Safety analysis of combination therapy

In terms of safety, this meta-analysis showed that the overall incidence of adverse events (*RR* = 0.52) and the incidence of breast tenderness (*RR* = 0.44) were both lower in the combination therapy group than in the low-dose FMT alone group.

There was no statistically significant difference in endometrial thickness between the two groups (*P* = 0.86), indicating that the combination therapy did not exert additional estrogen-like stimulation on the endometrium. However, the treatment duration in all included studies was ≤ 3 months, and the long-term safety of the combination therapy (e.g., endometrial hyperplasia, breast cancer risk, thromboembolism) remains unknown. Accordingly, the current findings only suggest favorable short-term tolerability of the combination therapy, and long-term monitoring is still needed in clinical practice.

### 4.5 Publication bias analysis

Regarding the assessment of publication bias, Egger’s test for the FSH outcome did not detect significant publication bias (*P* > 0.05). However, the number of included studies for this outcome was exactly at the lower recommended threshold, which may limit statistical power and cannot completely rule out the possibility of publication bias. For the remaining outcomes (e.g., E_2_, LH, lipid parameters), fewer than 10 studies were included, and thus formal publication bias tests were not performed. Consequently, the possibility of unpublished negative results cannot be excluded, and the therapeutic effect of the combination therapy may be somewhat overestimated.

### 4.6 Analysis of sources of heterogeneity

In this meta-analysis, moderate to substantial heterogeneity was observed for several outcomes. This heterogeneity may reflect clinical diversity (e.g., differences in symptom duration, age, and treatment duration across studies) as well as methodological diversity (e.g., none of the included studies explicitly reported allocation concealment or blinding).

To explore potential sources of heterogeneity, prespecified subgroup analyses were performed based on mean age (≥ 50 years vs. < 50 years) and treatment duration (≥ 3 months vs. < 3 months). For sex hormone outcomes (E_2_, FSH, and LH), neither age nor treatment duration could account for the observed heterogeneity.

Regarding lipid parameters, no statistically significant differences were observed between treatment duration subgroups for TC, TG, and LDL-C. Age appeared to be a partial source of heterogeneity for TC, TG, and LDL-C; however, the number of studies included in each subgroup was small, confidence intervals were wide, and statistical power was severely limited. Consequently, these findings are unstable and cannot be used as a basis for individualized treatment decisions.

Notably, for endometrial thickness, both age and treatment duration showed significant subgroup interactions (*P* < 0.00001). However, we observed a rare qualitative interaction (i.e., opposite directions of effect across subgroups): in the subgroups with mean age ≥ 50 years and treatment duration < 3 months, the combination therapy was associated with increased endometrial thickness (*MD* = 0.88), whereas in the subgroups with mean age < 50 years and treatment duration ≥ 3 months, it was associated with decreased endometrial thickness (*MD* = −0.33). Yusuf et al. (36) pointed out that such opposite-direction subgroup findings should generally not be believed, and that the overall result is more reliable than subgroup results. In this study, the overall analysis showed no significant difference in endometrial thickness between the two groups (*MD* = 0.09, *P* = 0.86). These subgroup findings are likely spurious due to small sample sizes and susceptibility to chance. Therefore, clinical decisions should be based on the overall effect and should not be influenced by these subgroup results.

In summary, the above subgroup findings should be interpreted with caution. First, all age-based subgroup analyses were performed using study-level weighted mean ages rather than individual patient data. Group means do not accurately reflect individual age distributions, and inferring individual-level treatment effects from such data is prone to ecological fallacy (37). Therefore, age-related subgroup results should only be used to explore sources of heterogeneity. Second, some subgroups contained very few studies, resulting in insufficient statistical power and inability to estimate within-subgroup heterogeneity; the results are thus susceptible to the influence of a single study. Taken together, all subgroup analyses should be considered exploratory and should not be used as a basis for interpreting the main findings.

### 4.7 Limitations of this study

Several limitations of this study should be acknowledged.

1. The 10 included RCTs did not adequately report allocation concealment or blinding of participants and personnel. A small number of studies only mentioned “randomization” without specifying the method of random sequence generation. These design flaws may have led to overestimation of the treatment effects (38).

2. All included studies were conducted in China, representing a single source of evidence. The lack of international multicenter data introduces potential geographical bias, which seriously limits the generalizability of our findings.

3. None of the included studies reported long-term follow-up data, precluding assessment of the long-term efficacy and safety of the combination therapy.

4. Substantial heterogeneity was observed for several outcomes. Although subgroup analyses by age and treatment duration were performed, the sources of heterogeneity could not be fully explained, which may affect the reliability of the conclusions.

5. The GRADE certainty of evidence was rated as low for most outcomes, reducing the credibility of the meta-analysis results.

6. Subgroup analyses were performed based on aggregated study-level data rather than individual patient data, which may introduce aggregation bias (39).

### 4.8 Recommendations for future clinical RCTs

Based on the limitations of this study and the current state of research on integrated traditional Chinese and Western medicine for PMS, the following recommendations are proposed for future high-quality RCTs:

1. Strictly adhere to the CONSORT 2025 statement to conduct well-designed and transparently reported RCTs (40). The methods of random sequence generation, allocation concealment, and blinding should be clearly described to provide more robust clinical evidence for Kuntai capsule combined with low-dose Femoston.
2. Encourage the conduct of international multicenter, large-sample studies to enhance the generalizability of the results.
3. Future studies should add follow-up assessments and systematically document adverse events to provide evidence on long-term safety.
4. Report baseline patient characteristics in detail, including body mass index (BMI), symptom duration, and perimenopausal stage (e.g., the STRAW + 10 staging system), to facilitate subgroup assessments and improve study reproducibility.
5. Design study protocols in accordance with the SPIRIT-TCM statement (41) and register them prospectively in trial registries (e.g., ClinicalTrials.gov or the Chinese Clinical Trial Registry) to enhance research transparency and credibility.

## 5 Conclusion

Current evidence suggests that KTC combined with low-dose FMT may confer benefits in ameliorating clinical symptoms, modulating sex hormone levels, and improving lipid profiles in patients with PMS, with a lower incidence of adverse events. However, the lipid benefit reflects only the overall analysis; the age-related subgroup differences are insufficient to guide clinical decisions due to lack of evidence, and should be considered hypothesis-generating findings only. They cannot be used to infer a true association between treatment effect and age.

Given the limited methodological quality and small sample sizes of the included studies, and the low to very low GRADE certainty of evidence for most outcomes, the confidence in these conclusions is limited. Well-designed, large-sample, high-quality randomized controlled trials are warranted to further validate the long-term efficacy and safety of the combination therapy and to inform rational clinical use.

## Data Availability

The datasets presented in this study can be found in online repositories. The names of the repository/repositories and accession number(s) can be found in the article/Supplementary material.

## Footnotes

1
https://www.gradepro.org

2
https://gdt.gradepro.org/app/handbook/handbook.html
