# Supplementary material for: Efficacy of Kuntai capsules with low-dose Femoston for perimenopausal syndrome: a meta-analysis
Source: Front Med (Lausanne). 2026 Jun 24;13:1768247. doi: 10.3389/fmed.2026.1768247 (PMC13341697; doi:10.3389/fmed.2026.1768247)
Supplement: Supplementary file 1 [file Table_1.docx]

Supplementary Material

Catalogue

[1. Search strategies 1](#_Toc31039)

[2. Table S1 3](#_Toc27338)

[3. Figure S1 3](#_Toc25393)

[4. Table S2 4](#_Toc23703)

[5. Table S3 4](#_Toc11139)

[6. Table S4 4](#_Toc9369)

[7. Table S5 4](#_Toc564)

[8. Table S6 5](#_Toc24446)

[9. Table S7 5](#_Toc13120)

[10. Table S8 5](#_Toc19274)

# 1. Search strategies

**Search strategy in Chinese databases**

1. **China National Knowledge Infrastructure (CNKI)**

（篇关摘：围绝经期综合（精确)）OR（篇关摘：围绝经期（精确)）OR（篇关摘：绝经期(精确)）OR（篇关摘：更年期(精确)）AND（篇关摘：坤泰胶囊(精确)）AND（篇关摘：芬吗通(精确)）OR（篇关摘：雌二醇/雌二醇地屈孕酮片(精确)）OR（篇关摘：雌孕激素复合制剂(精确)）OR(篇关摘：雌二醇地屈孕酮(精确)）OR（篇关摘：雌孕激素(精确) AND（摘要：随机(精确) OR （摘要：随机对照(精确)）

1. **China Wanfang Data**

题名或关键词:(围绝经期综合征 OR 更年期 OR 绝经期 OR 围绝经期) and 题名或关键词:(坤泰胶囊) and 题名或关键词:(芬吗通 OR 雌二醇/雌二醇地屈孕酮片 OR 雌孕激素复合制剂 OR 雌二醇地屈孕酮 OR 雌孕激素) and 题名或关键词:(随机 OR 随机对照)

1. **VIP Journal Database(VIP)**

题名或关键词=围绝经期综合征 OR 更年期 OR 绝经期 OR 围绝经期AND题名或关键词=坤泰胶囊 AND题名或关键词=芬吗通 OR 雌二醇/雌二醇地屈孕酮片 OR 雌孕激素复合制剂 OR 雌二醇地屈孕酮 OR 雌孕激素 AND题名或关键词=随机 OR 随机对照

1. **Chinese Biomedical Literature Database (CBM)**

("围绝经期"[不加权:扩展])) OR (("围绝经期综合征"[常用字段:智能] OR "更年期"[常用字段:智能] OR "绝经期"[常用字段:智能]) AND ("香芍颗粒"[常用字段:智能])

**Search strategy in English databases**

1. **Pubmed**

#1 "Perimenopause"[MeSH Terms]

#2 "Perimenopause"[Title/Abstract] OR "Climacteric"[Title/Abstract] OR "Menopause"[Title/Abstract]

#3 #1 OR #2

#4 "kuntai capsule"[Title/Abstract]

#5 "femoston"[Title/Abstract] OR ("estradiol"[Title/Abstract] AND "dydrogesterone tablets"[Title/Abstract])

#6 "Randomized Controlled Trial"[Publication Type]

#7 "random"[Title/Abstract] OR "blind"[Title/Abstract] OR "random*"[Title/Abstract]

#8 #6 OR #7

#9 #3 AND #4 AND #5 AND #8

(((("Perimenopause"[Mesh]) OR (((Perimenopause[Title/Abstract]) OR (Climacteric[Title/Abstract])) OR (Menopause[Title/Abstract]))) AND (kuntai capsule[Title/Abstract])) AND ((femoston[Title/Abstract]) OR (Estradiol[Title/Abstract] AND Dydrogesterone Tablets[Title/Abstract]))) AND (("Randomized Controlled Trial" [Publication Type]) OR (((random[Title/Abstract]) OR (blind[Title/Abstract])) OR (random*[Title/Abstract])))

1. **Embase**

#1 'perimenopause'/exp OR perimenopause

#2 'climacteric':ab,ti OR 'menopause':ab,ti

#3 #1 OR #2

#4 kuntai AND capsule

#5 femoston

#6 randomized AND controlled AND trial

#7 'random':ab,ti OR 'random*':ab,ti OR 'blind':ab,ti

#8 #6 OR #7

#9 #3 AND #4 AND #5 AND #8

1. **Cochrane library**

#1 Perimenopause

#2 (Climacteric):ti,ab,kw OR (Menopause):ti,ab,kw

#3 #1 OR #2

#4 kuntai capsule

#5 femoston

#6 randomized controlled trial

#7 (random):ti,ab,kw OR (random*):ti,ab,kw OR (blind):ti,ab,kw

#8 #6 OR #7

#9 #3 AND #4 AND #5 AND #8

1. **Web of science**

#1 TS=(Perimenopause or Climacteric or Menopause)

#2 TS=(kuntai capsule)

#3 TS=(femoston)

#4 TS=(randomized controlled trial or random or random* or blind)

#5 #1 AND #2 AND #3 #4

# **2. Table S1**

Table S1 Weighted mean age of participants in included studies

| Study | Sample size(n) | | | Age (years) | | | Mean age |
| --- | --- | --- | --- | --- | --- | --- | --- |
|  | T | C | | T | C | |  |
| Cai 2022 | 41 | | 40 | 49.5±3.2 | | 48.8±3.6 | 49.15 |
| [Meng 2024](#Meng) | 40 | | 40 | 48.71±1.94 | | 49.15±2.57 | 48.93 |
| [Zhang 2024](#Zhang) | 81 | | 81 | 47.45±4.21 | | 47.1±3.97 | 47.27 |
| [Ke 2023](#Ke) | 47 | | 47 | 48.1±4.8 | | 47.9±4.6 | 48.00 |
| [Suo 2023](#Suo) | 49 | | 49 | 51.32±2.17 | | 51.27±2.19 | 45.50 |
| [Gao 2022](#Gao) | 50 | | 50 | 45.46±2.33 | | 45.54±2.31 | 51.31 |
| [Lu 2020](#Lu) | 35 | | 35 | 51.27±3.31 | | 51.35±3.42 | 51.29 |
| [Guo 2019](#Guo) | 71 | | 71 | 52.26±3.24 | | 53.45±3.36 | 48.19 |
| [Fu 2019](#Fu) | 73 | | 73 | 47.98±4.92 | | 48.41±4.77 | 52.85 |
| [Wu 2019](#Wu) | 42 | | 42 | 51.84±3.54 | | 52.03±3.73 | 51.93 |

Note: T: Treatment group, C: Control group. The weighted mean age was calculated using the formula recommended by the Cochrane Handbook: (n1 × mean1 + n2 × mean2) / (n1 + n2), where n1 and n2 are the sample sizes of the treatment and control groups, respectively, and mean1 and mean2 are the corresponding mean ages.

# **3. Figure S1**


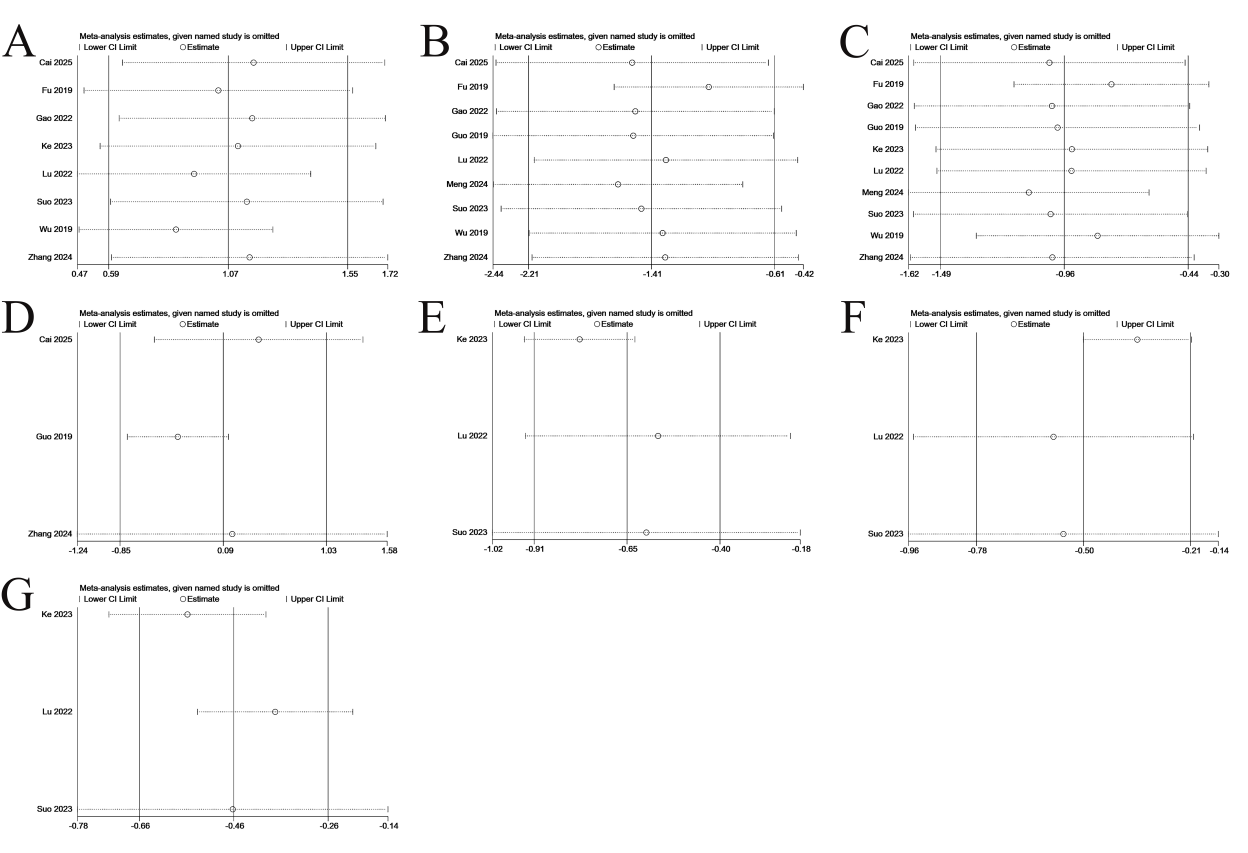


Figure S1 Summary of leave‑one‑out sensitivity analysis for outcomes with substantial heterogeneity (I² ≥ 50%).

Note:Each circle represents the pooled effect size after omitting one individual study. The horizontal lines indicate the 95% confidence interval (*CI*). The vertical solid line represents the overall pooled effect size including all studies. Panels (A) E2, (B) LH, (C) FSH, (D) endometrial thickness, (E) TC, (F) LDL‑C, (G) TG. SMD analysis for E_2_, LH, FSH; MD analysis for endometrial thickness and lipids.

# **4. Table S2**

Table S2 Leave‑one‑out sensitivity analysis for E_2_ levels.

| Study omitted | Heterogeneity analysis | | Meta analysis | | | |
| --- | --- | --- | --- | --- | --- | --- |
|  | *I^2^*（%） | *P* value | *SMD* | [95% Conf. Interval] | | *P* value |
| Cai 2025 | 91 | <0.00001 | 1.16 | 0.64 | 1.69 | <0.0001 |
| Fu 2019 | 91 | <0.00001 | 1.02 | 0.49 | 1.56 | 0.0002 |
| Gao 2022 | 91 | <0.00001 | 1.16 | 0.63 | 1.69 | <0.0001 |
| Ke 2023 | 92 | <0.00001 | 1.10 | 0.55 | 1.65 | <0.0001 |
| Lu 2022 | 89 | <0.00001 | 0.93 | 0.46 | 1.39 | <0.0001 |
| Suo 2023 | 91 | <0.00001 | 1.14 | 0.59 | 1.68 | <0.0001 |
| Wu 2019 | 84 | <0.00001 | 0.86 | 0.47 | 1.24 | <0.0001 |
| Zhang 2024 | 91 | <0.00001 | 1.15 | 0.60 | 1.70 | <0.0001 |

# **5. Table S3**

Table S3 Leave‑one‑out sensitivity analysis for LH levels.

| Study omitted | Heterogeneity analysis | | Meta analysis | | | |
| --- | --- | --- | --- | --- | --- | --- |
|  | *I^2^*（%） | *P* value | *SMD* | [95% Conf. Interval] | | *P* value |
| Cai 2025 | 97 | <0.00001 | -1.52 | -2.40 | -0.64 | 0.0007 |
| Fu 2019 | 94 | <0.00001 | -1.02 | -1.64 | -0.41 | 0.001 |
| Gao 2022 | 97 | <0.00001 | -1.50 | -2.40 | -0.60 | 0.001 |
| Guo 2019 | 97 | <0.00001 | -1.51 | -2.42 | -0.61 | 0.001 |
| Lu 2022 | 97 | <0.00001 | -1.30 | -2.15 | -0.45 | 0.003 |
| Meng 2024 | 96 | <0.00001 | -1.61 | -2.42 | -0.80 | <0.0001 |
| Suo 2023 | 97 | <0.00001 | -1.46 | -2.37 | -0.55 | 0.002 |
| Wu 2019 | 97 | <0.00001 | -1.32 | -2.19 | -0.46 | 0.003 |
| Zhang 2024 | 97 | <0.00001 | -1.31 | -2.17 | -0.45 | 0.003 |

# **6. Table S4**

Table S4 Leave‑one‑out sensitivity analysis for FSH levels.

| Study omitted | Heterogeneity analysis | | Meta analysis | | | |
| --- | --- | --- | --- | --- | --- | --- |
|  | *I^2^*（%） | *P* value | *SMD* | [95% Conf. Interval] | | *P* value |
| Cai 2025 | 94 | <0.00001 | -1.02 | -1.59 | -0.44 | 0.0005 |
| Fu 2019 | 89 | <0.00001 | -0.75 | -1.16 | -0.34 | 0.0003 |
| Gao 2022 | 94 | <0.00001 | -1.01 | -1.59 | -0.43 | 0.0007 |
| Guo 2019 | 94 | <0.00001 | -0.98 | -1.58 | -0.38 | 0.001 |
| Ke 2023 | 94 | <0.00001 | -0.92 | -1.49 | -0.35 | 0.002 |
| Lu 2022 | 94 | <0.00001 | -0.92 | -1.49 | -0.36 | 0.001 |
| Meng 2024 | 93 | <0.00001 | -1.10 | -1.61 | -0.60 | <0.0001 |
| Suo 2023 | 94 | <0.00001 | -1.01 | -1.59 | -0.43 | 0.0006 |
| Wu 2019 | 93 | <0.00001 | -0.81 | -1.33 | -0.30 | 0.002 |
| Zhang 2024 | 94 | <0.00001 | -1.00 | -1.60 | -0.41 | 0.001 |

# **7. Table S5**

Table S5 Leave‑one‑out sensitivity analysis for endometrial thickness.

| Study omitted | Heterogeneity analysis | | Meta analysis | | | |
| --- | --- | --- | --- | --- | --- | --- |
|  | *I^2^*（%） | *P* value | *MD* | [95% Conf. Interval] | | *P* value |
| Cai 2025 | 95 | <0.00001 | 0.41 | -0.54 | 1.36 | 0.40 |
| Guo 2019 | 70 | 0.07 | -0.33 | -0.79 | 0.13 | 0.16 |
| Zhang 2024 | 98 | <0.00001 | 0.17 | -1.24 | 1.58 | 0.82 |

# **8. Table S6**

Table S6 Leave‑one‑out sensitivity analysis for TC levels.

| Study omitted | Heterogeneity analysis | | Meta analysis | | | |
| --- | --- | --- | --- | --- | --- | --- |
|  | *I^2^*（%） | *P* value | *MD* | [95% Conf. Interval] | | *P* value |
| Ke 2023 | 0 | 0.70 | -0.78 | -0.93 | -0.63 | <0.00001 |
| Lu 2022 | 79 | 0.03 | -0.57 | -0.93 | -0.21 | 0.002 |
| Suo 2023 | 86 | 0.008 | -0.60 | -1.02 | -0.18 | 0.005 |

# **9. Table S7**

Table S7 Leave‑one‑out sensitivity analysis for TG levels.

| Study omitted | Heterogeneity analysis | | Meta analysis | | | |
| --- | --- | --- | --- | --- | --- | --- |
|  | *I^2^*（%） | *P* value | *MD* | [95% Conf. Interval] | | *P* value |
| Ke 2023 | 0 | 0.34 | -0.56 | -0.72 | -0.39 | <0.00001 |
| Lu 2022 | 35 | 0.22 | -0.37 | -0.53 | -0.21 | <0.00001 |
| Suo 2023 | 81 | 0.02 | -0.46 | -0.78 | -0.14 | 0.005 |

# 10. Table S8

Table S8 Leave‑one‑out sensitivity analysis for LDL-C levels.

| Study omitted | Heterogeneity analysis | | Meta analysis | | | |
| --- | --- | --- | --- | --- | --- | --- |
|  | *I^2^*（%） | *P* value | *MD* | [95% Conf. Interval] | | *P* value |
| Ke 2023 | 0 | 0.79 | -0.36 | -0.50 | -0.21 | <0.00001 |
| Lu 2022 | 86 | 0.008 | -0.58 | -0.95 | -0.21 | 0.002 |
| Suo 2023 | 91 | 0.0008 | -0.55 | -0.96 | -0.14 | 0.009 |
